# Supplementary material for: Stimulus-Responsiveness of Thermo-Sensitive Polymer Hybridized with N-Doped Carbon Quantum Dots and Its Applications in Solvent Recognition and Fe3+ Ion Detection
Source: Polymers (Basel). 2022 May 12;14(10):1970. doi: 10.3390/polym14101970 (PMC9147206; doi:10.3390/polym14101970)
Supplement: Supplementary file 1 [file polymers-14-01970-s001.zip › polymers-1712957-supplementary.pdf]

# Stimulus-Responsiveness of Thermo-Sensitive Polymer Hybridized with N-Doped Carbon Quantum Dots and Its Applications in Solvent Recognition and $Fe^{3+}$ Ion Detection

**Tong Chen, Hongwei Zhang and Sanping Zhao ***

State Key Laboratory of New Textile Materials and Advanced Processing Technologies, Wuhan Textile University, Wuhan 430073, China; chengtong1997518@163.com (T.C.); hwzhang@wtu.edu.cn (H.Z.); * Correspondence: 2006184@wtu.edu.cn; Tel./Fax:+86-27-8742-6559

# Supporting information

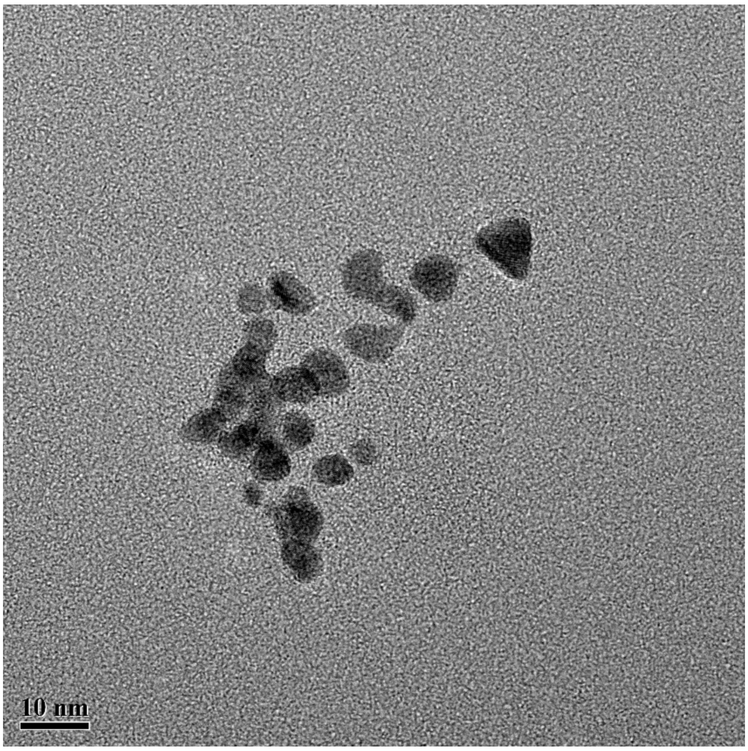


**Figure S1.** TEM images of $poly_{14}$-N-CQDs.

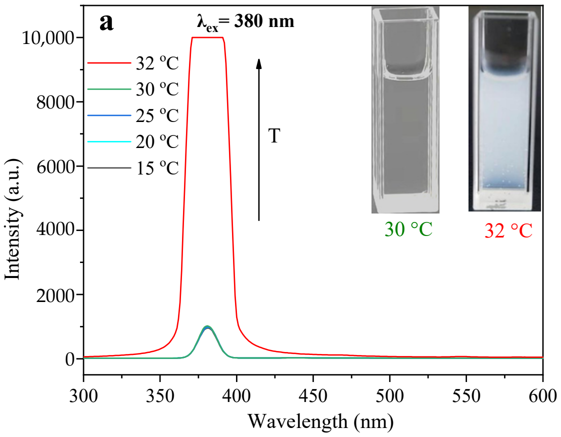


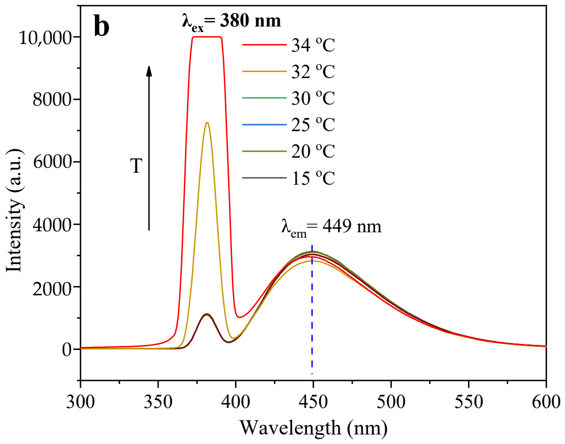


**Figure S2.** The fluorescence emission spectra of the poly$_{14}$ (a) and the mixture of poly$_{14}$ and N-CQDs (b) at 380 nm excitation wavelength at different temperatures.

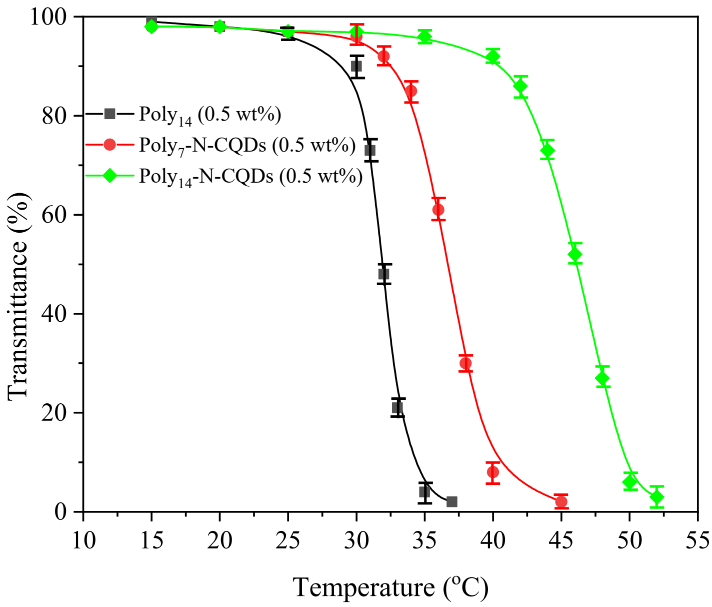


**Figure S3.** The transmittance of different samples with a concentration of 0.5 wt% as a function of the temperature.

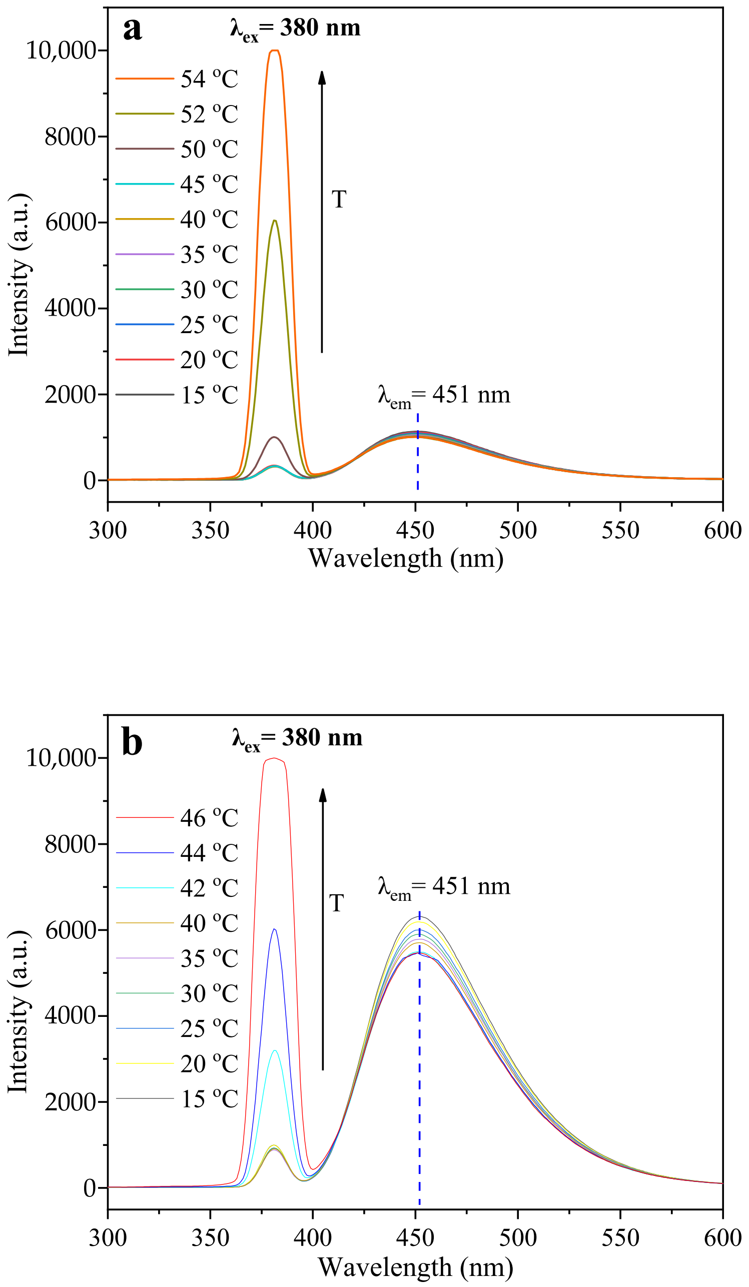


**Figure S4.** The fluorescence emission spectra of 0.1 wt% (a) and 1 wt % (b) of poly$_{14}$-N-CQDs solutions at 380 nm excitation wavelength and different temperatures.

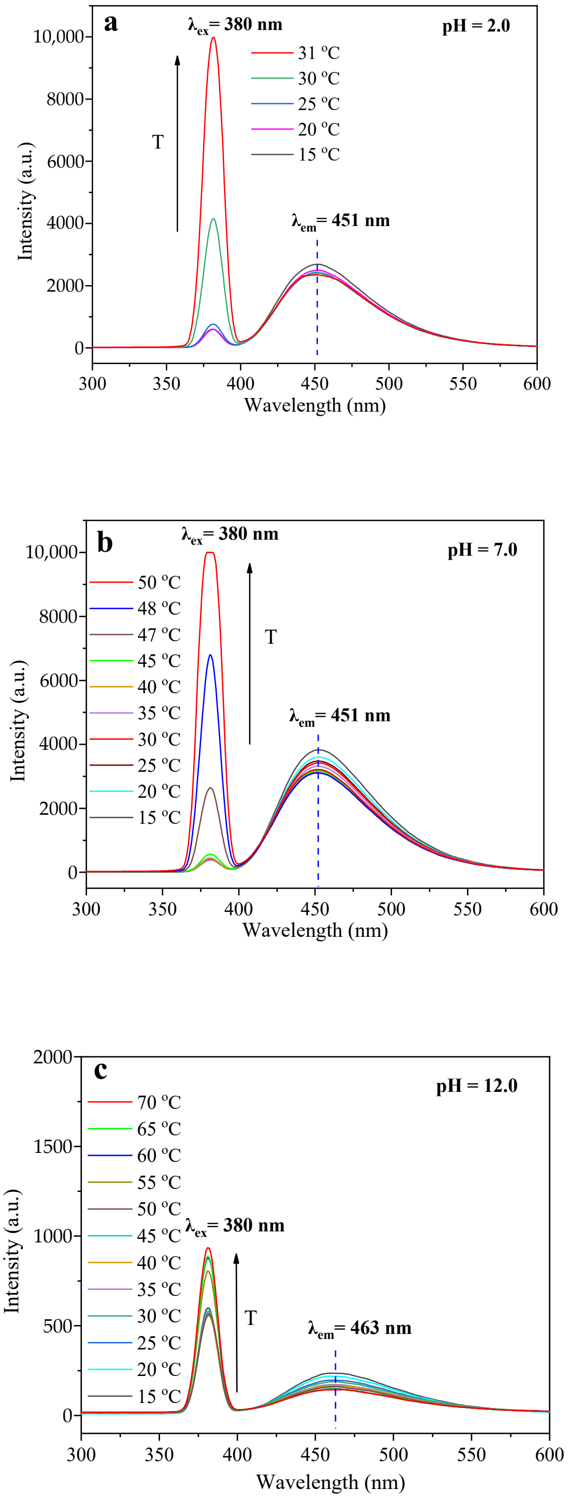


**Figure S5.** The fluorescence emission spectra of poly-N-CQDs at pH 2.0 (a), pH 7.0 (b) and pH 12.0 (c) at 380 nm excitation wavelength and different temperatures.

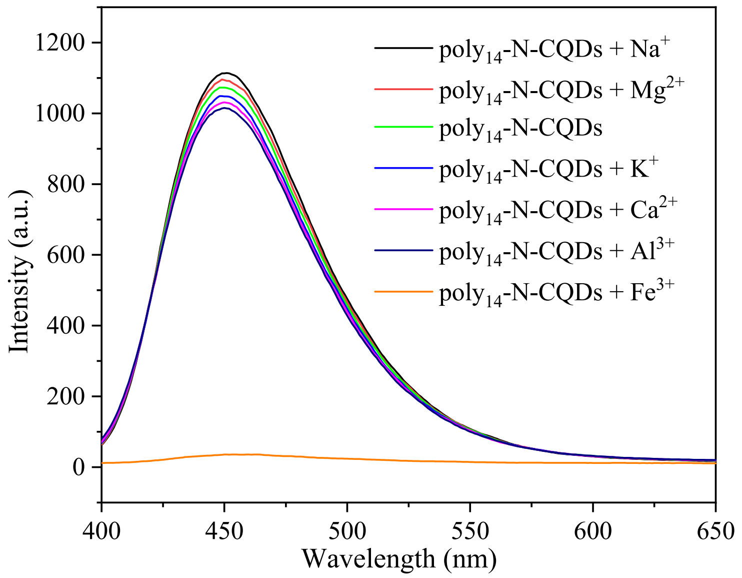


**Figure S6.** The fluorescence emission spectra of 0.1 wt% $poly_{14}$-N-CQDs solution in the presence of different metal ions (4 mM) at 380 nm excitation wavelength.
